# Interventions to improve access to care for abnormal uterine bleeding: A systematic scoping review

**DOI:** 10.1002/ijgo.14224

**Published:** 2022-05-05

**Authors:** Parimala Suganthini Kanagasabai, Sara Filoche, Rebecca Grainger, Claire Henry, Jean Hay‐Smith

**Affiliations:** ^1^ Department of Obstetrics, Gynaecology and Women's Health University of Otago Wellington Wellington New Zealand; ^2^ Department of Medicine University of Otago Wellington Wellington New Zealand; ^3^ Rehabilitation Teaching and Research Institute University of Otago Wellington Wellington New Zealand

**Keywords:** abnormal uterine bleeding, accessibility, health care access, systematic scoping review

## Abstract

**Background:**

Women with abnormal uterine bleeding (AUB) experience barriers to accessing healthcare services.

**Objectives:**

To identify and describe the evidence on interventions to improve healthcare access of women with AUB.

**Search strategy:**

A systematic search of databases including Medline, CINAHL, EMBASE, Scopus, and Cochrane register for clinical trials on February 26, 2021.

**Selection criteria:**

Studies including women with AUB and investigating an intervention to improve access at the levels of individual patient, community, organization, health system, or medical education.

**Data collection and analysis:**

Data extraction and descriptive analysis of the country, study design, settings, participant characteristics, intervention, outcome measures, and key findings.

**Main results:**

We identified 20 studies and most interventions (13 studies) targeted organizational changes. Creating a multidisciplinary team, bringing services together and developing a care pathway improved the availability of services. Management of AUB in an outpatient setting improved the affordability. The use of decision aids improved patient engagement in consultations. There is a lack of interventions at an individual or community level targeting health literacy, health beliefs, social acceptability, and opportunity to reach and pay for services.

**Conclusions:**

Community‐based culturally‐adapted interventions focusing on access to women with different socio‐economic and cultural backgrounds should be investigated.

## 1 BACKGROUND

Abnormal uterine bleeding (AUB), is the term used to describe any change from normal menstruation or a normal menstrual cycle pattern, including changes in the regularity, frequency, heaviness, or duration of blood flow of the normal menstrual pattern.^1^ The prevalence of AUB is common, affecting 10%–30% of women, and a spectrum of underlying conditions related to structure (endometrial cancer, polyps, fibroids, pregnancy complication), hormonal function (menopause) and contraceptive methods contribute to AUB.^2^ Abnormal bleeding can reduce quality of life. Women often experience AUB symptoms for years before seeking care or receiving treatment.^3^, ^4^, ^5^, ^6^ In a UK national audit including 14 545 women with heavy menstrual bleeding (HMB) attending secondary care, 74% of women had symptoms for more than 1 year before seeking treatment and 30.4% reported no previous treatment in primary care.^5^ A multinational survey across women with HMB in Canada, the USA, Brazil, France, and Russia showed that the mean time from first symptoms to seeking help was 2.9 (±3.1) years. Forty percent of women had not seen a health care professional about their HMB. Furthermore, over half (54%) had never been diagnosed or treated and only 20% had been diagnosed and received appropriate treatment.^6^ Some of the barriers to healthcare access identified include taboo or stigma that prevented women from disclosing menstrual problems, embarrassment from exposing private body parts, discomfort with gynecologic examination, and fear of the possible diagnosis or gaps in health literacy resulting in normalizations of symptoms. Lack of trust, rapport, and shared decision making in doctor‐patient relationships can make communication about sensitive gynecologic issues difficult. For women who did engage with primary healthcare providers, dismissal of symptoms^4^ and health providers' lack of procedural skills for AUB management were problematic.^7^ Health system issues like long waiting lists and delays in assessment and diagnosis were also identified.^4^ Barriers to access could lead to delayed management and racial disparities in gynecologic examinations, diagnosis, and healthcare outcomes.^8^ Interventions or programs that address the barriers and health needs of women may be able to improve the access to health care for women with AUB, and then improve outcomes.

Levesque et al.^9^ define access to health care as the opportunity to reach and obtain appropriate healthcare services in situations of perceived need for care. Access is considered to be dependent on features of health systems, organizations, and providers; and also on features of the population such as characteristics of individuals, households, and physical and social environments. Based on Levesque et al.'s literature‐informed conceptual framework, accessibility to health services is categorized into five dimensions comprising approachability, acceptability, availability and accommodation, affordability, and appropriateness. Five corresponding abilities of populations that interact with the dimensions of accessibility include the ability to perceive, ability to seek, ability to reach, ability to pay, and ability to engage. The aim of this systematic scoping review is to identify and describe the evidence on the effectiveness of interventions to improve healthcare access of women with AUB. As access is multifactorial, this review focuses on interventions addressing factors at personal, household, community, population, and health system levels that target improving access for women with AUB.

## 2 MATERIALS AND METHODS

A scoping review is an appropriate methodology for reviewing large bodies of literature to generate an overview of research on a topic. It determines the range of studies that are available, summarizes research results, and identifies evidence gaps. Our scoping review methods were based on the six stages described by Arksey and O'Malley^10^: (1) identifying the research question; (2) identifying relevant studies; (3) selecting studies; (4) charting the data; (5) collating, summarizing, and reporting the results; and (6) consulting with relevant stakeholders. We reported the scoping review based on the Preferred Reporting Items for Systematic Review and Meta‐Analysis (PRISMA) guidelines—extension for scoping review.^11^

### 2.1 Identifying the research question

Our previous review identified barriers to healthcare access for women with AUB.^3^ Our qualitative study then provided further insights into barriers to women's journey of care for AUB at a tertiary medical center in New Zealand.^4^ Before planning an intervention aimed to improve access to care for AUB, we proposed a scoping review to examine the existing evidence for such interventions and identify gaps in the intervention literature. The scoping review protocol is available on request. The review addresses the following research questions: (1) What are the types of population or AUB conditions included in the studies? (2) In which settings are these interventions provided? (3) Which types of studies are available in the literature? (4) What type of interventions or programs have been successful or unsuccessful in improving access to care for women with AUB? (5) What access‐related outcome measures have been used?

### 2.2 Identifying relevant studies

We conducted a systematic search of Medline, CINAHL, EMBASE, Scopus, and Cochrane register database for clinical trials. The search was conducted from database inception to February 26, 2021. The search strategy was developed by three of the authors (PSK, CH, and SF) and validated by a reference librarian. To identify studies on AUB, we included the search terms used for abnormal uterine bleeding as indicated by FIGO (the International Federation for Gynecology & Obstetrics).^2^ To search for studies on access to health care, we used terms based on the conceptual framework of “Patient‐centred Access to Health Care”.^9^ These included terms related to health services dimensions and population dimensions. The search strategy and specific search terms are provided in a supplementary online file (Appendix S1).

Our librarian executed the search strategy and provided data to the primary author (PSK) as a compressed Endnote library.

### 2.3 Study selection

A study was included if it: (1) included patients (service users) with AUB with any underlying cause; (2) involved healthcare/service providers, health administrators, or support staff as participants; (3) was an intervention or program evaluation study with quantitative, qualitative, or mixed‐method study methodology; (4) included interventions or programs at an individual patient level, health provider level, administration level, system level (policies) or medical education level; and (5) was in English. Exclusion criteria included studies investigating diagnostic, medical, or surgical interventions/procedures. We also excluded opinion pieces, commentary, letters, and theses.

Using the eligibility criteria, the primary author (PSK) performed the title and abstract screening. At the full‐text screening stage, another author (SF) was involved to discuss articles for inclusion in the final review. Additionally, this author (SF) reviewed a sample of 11 full‐text articles.

### 2.4 Data collection/Charting the data

A data collection instrument (spreadsheet) was used to extract the study characteristics of included studies. Data were extracted under the following headings: author, year of publication, country of study, study design, participant characteristics and study settings, intervention, access‐related outcome measure, and key findings related to either the effect of an intervention (quantitative studies) or the experience of intervention (qualitative studies). Charting was an iterative process and the data collection form was categorized or sectioned based on the type of interventions.

### 2.5 Data summary and synthesis of results

Data obtained were synthesized to map the research evidence available and provide information on literature, particularly the main type of interventions available and intervention settings. We grouped the data based on the type of intervention and the dimensions of access according to Levesque et al.^9^ Health services‐based interventions are at an organizational, policies, or health‐provider level. Population‐based interventions could be programs at an individual patient, household, or community level.

### 2.6 Optional consultation

We did not perform any consultation with stakeholders because of time constraints.

## 3 RESULTS

**FIGURE 1:**
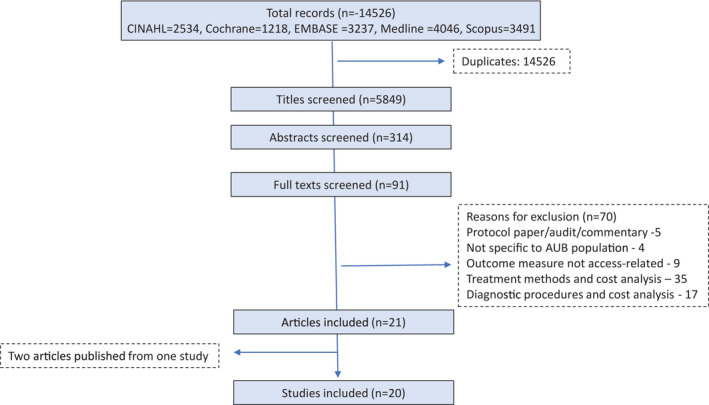
Flowchart of study selection

Our search resulted in 14 526 records with 5849 remaining after the removal of duplicates. Figure 1 illustrates the selection process. Following title screening, we included 314 records, which was reduced to 91 after abstract screening. After full‐text screening, 21 papers^12^, ^13^, ^14^, ^15^, ^16^, ^17^, ^18^, ^19^, ^20^, ^21^, ^22^, ^23^, ^24^, ^25^, ^26^, ^27^, ^28^, ^29^, ^30^, ^31^, ^32^, ^33^ were included. Two papers^29^, ^30^ were from one study, so the total number of included studies was 20. We have cited Vuorma et al.^29^ to represent both papers. Reasons for exclusion at full‐text screening were the type of publication (e.g. protocol, commentary, conference proceedings), studies without access outcomes, and cost analyses of diagnostic or treatment interventions. Cost analysis studies (52 in total) compared the cost‐effectiveness of various medical or surgical treatments and diagnostic procedures rather than financial interventions to improve accessibility such as providing incentives or capitation fees to patients. Table 1 provides the description of included studies. Dimensions of access targeted by included studies are provided in Table 2.

**TABLE 1:** Data extraction of included studies

First author/ Year, place	Study type	Population/ Setting	Intervention/ Sample size	Access outcome measures	Instrument	Findings
Organizational interventions (Creating multidisciplinary team)						
Wygant^12^ 2019, USA	Quasi‐experimental	AUB Academic center	Collaborative hysteroscopy clinic *n* = 647	Availability *(Waiting time for appointment, number of appointment slots)*	Patient medical records	The appointment slots in the intervention period increased from 423 to 753 (57.5%). Waiting time decreased with 63% of patients scheduled within 0–13 days of initial contact compared with 30% of patients before intervention.
Organizational interventions (Bringing services together)						
Lotfallah^13^ 2005, UK	Retrospective	PMB General Hospital	One‐stop clinic *n* = 308	Availability *(Number of patients managed in first visit)*	Patient records	With intervention 216/308 (70%) of patients were totally managed and discharged at the first visit and hospital admission was avoided in 258/308 (83.7%) of cases
Sulaiman^14^ 2004, UK	Retrospective	PMB General Hospital	One‐stop postmenopausal clinic *n* = 95	Availability *(Waiting times, number of visits to hospital, treatment time from first consultation to a management plan)*	Patient charts	Time from referral to first consultation was shorter in the PMB clinic and the women had fewer visits. In the PMB clinic, 68/95 (72%) women received immediate reassurance and were discharged after the first consultation.
Mohammad^15^ 2003, UK	Prospective	PMB General Hospital	One‐stop PMB clinic n = 80	Availability *(Referral time, waiting time, number of patients managed in a single visit)* Appropriateness *(Satisfaction with service)*	A bespoke form in patient records	At PMB clinic, the mean time from GP referral to consultation was 14.8 days (range 2–24 days), 71/80 (89%) patients were managed in a single visit and 70/80 (87.5%) women considered the clinic visit satisfactory.
Abu^16^ 2001, UK	Prospective	AUB General Hospital	One‐stop menstrual clinic Survey *n* = 98 Interview *n* = 22	Ability to engage *(patient information)* Appropriateness *(coordination, continuity)* Availability *(waiting for appointment)* Acceptability *(choice of doctor)*	Patient career diary Survey questionnaire Qualitative interviews	Significant positive experience with one‐stop menstrual clinic on all domains. Qualitative interview confirmed one‐stop clinic was more suited to the needs of patients
Dueholm^17^ 1999, Denmark	Prospective	AUB with BD General Hospital	One‐stop clinic *n* = 114	Availability *(number of patients receiving diagnosis and treatment plan in first visit)*	Not reported	A sufficient diagnosis of the uterine cavity was attained in 106/114 (93%) patients and in 73% of patients a treatment plan could be formulated.
Atiomo^18^ 1998, UK	Retrospective	PMB General Hospital	One‐stop clinic *n* = 212	Availability *(waiting time between referral and appointment, number of hospital visits)*	Review of records	In one visit,144/212 (67.9%) women were evaluated and reassured. Reduced number of visits for diagnosis and waiting period.
Organizational interventions (Continuity of care)						
Julian^19^ 2007 UK	Prospective	HMB Teaching hospital and general practices with one primary care trust	GP‐led Bridges pathway *n* = 99	Availability (*waiting time, fitting with appointments)* Acceptability *(choice of doctor)* Appropriateness *(coordination, continuity)*	Patient carrier diary	Bridges improved patient information, fitting in with appointments, choice of doctor, waiting time, and less “limbo” or patient experience of non‐coordination between primary and secondary care.
Organizational interventions (procedure in outpatient setting)						
Bennett^20^ 2020, Canada	Retrospective	AUB Women's hospital	Outpatient uterine assessment and treatment unit (UATU) *n* = 200	Affordability *(total cost)* Availability *(time savings per patient)*	Retrospective chart review (between April 1, 2014, and March 31, 2017)	Compared with usual care, care in the UATU was associated with a decrease in overall cost and a decrease in overall time to treatment
Diwakar^21^ 2016, UK	RCT	AUB General hospitals	Outpatient polyp treatment *n* = 254	Affordability *(cost utility of treatment)*	Questionnaires on treatment costs and out‐of‐pocket costs.	Outpatient treatment of uterine polyps appears to be more cost‐effective than inpatient treatment at willingness‐to‐pay thresholds.
Moawad^22^ 2014, USA	Retrospective	AUB Two academic centers	Office hysteroscopy *n* = 130	Affordability *(cost of treatment)*	Paper charts and electronic medical records	Conducting office hysteroscopy and then referring the patient for hysteroscopy in the operating room only if needed resulted in more cost savings when compared with operating room hysteroscopy only.
Ahonkallio^23^ 2012, Finland	Retrospective	HMB Teaching general hospital	Outpatient endometrial ablation *n* = 16	Affordability *(cost of an outpatient procedure with local anesthesia)*	Hospital's operative database and information from financial planning department	The outpatient procedure was €800 cheaper than the day‐case procedure for the health service provider.
Jack^24^ 2005, UK	RCT	HMB Teaching general hospital	Outpatient microwave endometrial ablation (MEA) *n* = 97	Affordability *(cost of treatment)*	Health service costs Costing questionnaires for non‐health costs	The mean health service costs were 95% lower for the patients in the post‐menses group outpatient MEA.
Patient‐based interventions (patient engagement in decision making)						
Aarts^25^ 2021, USA	Quasi‐experimental	HMB Academic center and community practices	Option grid encounter decision aid *n* = 32	Ability to engage *(shared decision making)*	Questionnaires Audio recorded Consultation report	Encountering decision aid during counseling resulted in greater levels of shared decision making both from a patient's and an observer's perspective.
Dietrich^26^ 2017, USA	Prospective	HMB with BD Children's Hospital	iPod Touch device with iperiod application *n* = 23	Ability to engage *(patient information, adherence to medicine)*	Patient information, compliance to medication and hospital admission	Improved compliance with medications and none admitted to the hospital due to missed medications.
Hess^27^ 2015, USA	RCT	AUB Women's Hospital	Preference elicitation and Adaptive conjoint analysis *n* = 183	Ability to engage *(shared decision making)*	Survey questionnaires at 6 weeks	A preference elicitation tool at the initial consultation visit did not reduce decision regret or improve treatment satisfaction among patients with AUB.
Protheroe^28^ 2007, UK	RCT (6 months follow up)	HMB 19 Primary care centers	The Clinical Guidance Tree computerized decision aid *n* = 74	Ability to engage *(shared decision making)*	Postal questionnaires	Significantly less decisional conflict and improved patient involvement in decision making in primary care.
Vuorma^29^, ^30^ 2003, 2004, Finland	RCT and prospective study	HMB Hospitals	Information booklet *n* = 184 (RCT) *n* = 206 (Cohort study)	Ability to engage *(Patient information, communication with staff)* Availability *(number of patients with planned treatment)* Affordability *(cost of treatment)*	Questionnaires Medical records	Treatment decision within 3 months was made more often in the intervention group than in the control group. No between‐group differences were detected in the change in anxiety, satisfaction or knowledge level. There were no marked disparities in treatment costs between groups.
Kennedy^31^ 2002, UK	RCT (2 years follow up)	HMB General hospitals	Booklet and videotape *n* = 296 Interview with preference elicitation *n* = 300	Ability to engage *(shared decision making)* Affordability *(cost of treatment)*	Questionnaires (satisfaction rating) At baseline, 6, 12 and 24 months	Interview group reported significantly higher opportunity for treatment decision making than control group. Cost savings with both interventions.
Community awareness						
Hossenbaccus^32^ 2021, Canada	Quasi‐experimental (pilot)	HMB with BD High schools	Let us talk period class presentation *n* = 161	Ability to perceive *(Knowledge of menorrhagia and bleeding disorder)*	Kahoot quizzes (online questionnaire). Feedback forms	Greater knowledge level and retention Students reported class as interesting, open, interactive, knowledgeable.

Abbreviations: AUB, abnormal uterine bleeding; BD, bleeding disorder; GP, general practitioner; HMB, heavy menstrual bleeding, PMB, postmenopausal bleeding; RCT, randomized controlled trial.

**TABLE 2:** Dimensions of access targeted by interventions in included studies

Healthcare services		Population, communities, individual	
Access dimensions	Reference	Access dimensions	Reference
Approachability		Ability to perceive	
Transparency	–	Health literacy	^32^
Outreach	–	Health beliefs	–
Information	–		
Screening	–		
Acceptability		Ability to seek	
Professional values	–	Personal values	–
Norms	–	Social values	–
Culture	–	Culture	–
Gender (choice of doctor)	16, 19	Gender	–
		Autonomy	–
Availability and accommodation		Ability to reach	
Geographic location	–	Living environments	–
Accommodation	–	Transport	–
Hours of opening	–	Mobility	–
Appointment mechanisms	12, 13, 14, 15, 16, 17, 18, 19, 20, 29	Social support	–
Affordability		Ability to pay	
Direct costs	20, 21, 22, 23, 24, 29, 31	Income	–
Indirect costs	–	Assets	–
Opportunity costs	–	Social capital	–
		Health insurance	–
Appropriateness		Ability to engage	
Technical quality	–	Empowerment	25, 27, 28, 29, 31
Interpersonal quality	–	Information	16, 26
Adequacy	–	Adherence	^26^
Coordination & continuity	15, 16, 19	Caregiver support	–

### 3.1 Description of study type, setting, and population

Ten studies^13^, ^14^, ^15^, ^16^, ^18^, ^19^, ^21^, ^24^, ^28^, ^31^ were conducted in the UK, five in the USA,^12^, ^22^, ^25^, ^26^, ^27^ two each in Finland^23^, ^29^ and Canada,^20^, ^32^ and one in Denmark.^17^ Seven studies^12^, ^16^, ^17^, ^20^, ^21^, ^22^, ^27^ included women with AUB, nine^19^, ^23^, ^24^, ^25^, ^26^, ^28^, ^29^, ^31^, ^32^ specifically included women with HMB, and four^13^, ^14^, ^15^, ^18^ included women with postmenopausal bleeding (PMB).

Thirteen studies were conducted in hospital settings, including 10 in general hospitals,^13^, ^14^, ^15^, ^16^, ^17^, ^18^, ^19^, ^21^, ^23^, ^24^ two at women's hospitals,^20^, ^27^ and one study at a children's hospital.^26^ Three studies^12^, ^22^, ^25^ recruited women from academic center clinics, of which one^25^ recruited women from community group practices as well. One study^19^ recruited women from general practices. One study was conducted solely in a primary care setting.^28^ One study recruited grade 9 girls from a school setting.^32^

There were 10 experimental studies, including three quasi‐experimental studies^12^, ^25^, ^32^ and seven randomized controlled studies.^20^, ^21^, ^24^, ^27^, ^28^, ^29^, ^31^ There were five prospective studies^15^, ^16^, ^17^, ^19^, ^26^ and six retrospective studies.^13^, ^14^, ^18^, ^20^, ^22^, ^23^ Two studies^16^, ^32^ included qualitative components (interviews/feedback from the participants).

### 3.2 Type of interventions

Thirteen of 20 studies investigated interventions to improve access that focused on changes at the healthcare service (organizational) level. Organizational interventions included one study creating a multidisciplinary team,^12^ six bringing services together,^13^, ^14^, ^15^, ^16^, ^17^, ^18^ one developing a care pathway,^19^ and five creating an outpatient setting for procedures.^20^, ^21^, ^22^, ^23^, ^24^ Population‐based interventions comprised six studies targeting patient education and engagement using decision aids for shared decision making in physician consultations.^25^, ^26^, ^27^, ^28^, ^29^, ^31^ A single study piloted an educational intervention to improve awareness among school children regarding AUB and bleeding disorders.^32^

### 3.3 Description and outcomes of health service‐based interventions

Studies investigating one‐stop menstrual or postmenopausal clinics where diagnosis and management services were co‐localized showed improvement in access measures including a reduced number of hospital visits, avoidance of hospital admissions, decreased waiting time for appointments, and increased coordination and continuity of care.^13^, ^14^, ^15^, ^16^, ^17^, ^18^ Collectively, these studies suggested that 68%–89% of patients were evaluated and managed in the first one‐stop clinic visit. A collaborative hysteroscopy clinic with women's health nurse and gynecologist improved service “availability” by increasing the number of appointment slots by more than 50% and reducing the waiting time for appointments.^12^ A prospective study, that interviewed 22 women with AUB, confirmed that a one‐stop clinic was suited to the needs of these women.^16^ A general practitioner‐led “Bridges pathway” for access to appropriate secondary care showed improvements in getting choice of appointment slot, choice of doctor, and coordination between primary and secondary care.^19^ This “Bridges pathway” involved the use of evidence‐based guidelines by the general practitioner for the management of AUB and access to booking for investigations and surgical treatment. Interventions such as changing a diagnostic procedure to the outpatient setting, creating an outpatient assessment and treatment unit, outpatient microwave endometrial ablation, outpatient polyp treatment, or office hysteroscopy, reduced the cost per patient when compared with procedures in the inpatient setting, and saved theater time.^20^, ^21^, ^22^, ^23^, ^24^

### 3.4 Description and outcomes of patient‐ or population‐based interventions

Interventions targeting patient information using bespoke interactive computerized decision aids such as “The clinical guidance tree”^28^ or “option grid encounter decision aid”^25^ improved shared decision making between physician and the patients. An iPod Touch device with access to period information, provider contact information for questions, and record details of menstrual cycles and medications improved compliance with medications resulting in fewer hospital days due to missed medications.^26^ Information booklets provided before the consultation resulted in quicker decision making in treatment.^29^, ^31^ One study showed cost savings in treatment with an information booklet, videotape, and preference elicitation (interview to elicit the preferences),^31^ but another study showed no cost savings in treatment with patient information booklet alone.^29^ Information booklet alone was found to make no difference to the anxiety, satisfaction, or knowledge level of women.^29^ A single community‐level pilot study “Let's talk periods” with 75 minutes of class presentation showed improved knowledge of menorrhagia and bleeding disorders.^32^

## 4 DISCUSSION

This scoping review identified 20 studies that aimed at improving aspects of access to care for women with AUB. Given the high prevalence of AUB in women across countries^2^ and consistent reporting of barriers for women with AUB to accessing care over the past two decades,^3^, ^5^, ^6^ it is concerning that there is such a limited number of intervention studies focusing on this topic.

Our results show that organizational interventions such as developing collaborative services appeared to improve the availability, coordination, and continuity of healthcare services for AUB provided in hospital settings. Assessment and management of AUB in an outpatient setting seemed to reduce service costs when compared with an inpatient setting. Patient engagement (shared decision making) in patient‐physician consultations improved with patient information booklets, videotapes, and computer‐based decision tools. There were no studies targeting other health service‐based access dimensions including approachability, acceptability, appropriateness and population‐based access dimensions such as the ability to perceive, seek, reach, and pay. Hence, many evidence gaps need to be addressed to enable research‐informed programs that can improve access to care and the quality of life for women with AUB.

This review found that intervention studies were commonly conducted in western or high‐income countries and in a hospital setting. We found only one study targeting availability and coordination of services at primary care.^19^ General practitioners in primary care are the first point of contact for patients with AUB and are important for early diagnosis of gynecologic conditions involving AUB.^4^, ^7^ More studies in the primary care setting are needed to support healthcare access for women with AUB in the community. No studies were identified at the health provider or medical education level. Interventions such as communication and interpersonal skill training have been shown to have a significant impact on empathic behavior for health professionals^33^ and such interventions have the potential to improve sensitivity towards menstrual issues. Furthermore, training general practitioners with procedural skills such as insertion of intrauterine devices and AUB guidelines would be helpful for the initial management of AUB.^5^ However, this requires increased resources, time, and financial costs.^34^

We did not find any study directly exploring acceptability, which includes cultural and social acceptance of services. Two studies did show improved satisfaction with choice of doctor with the one‐stop menstrual clinic^16^ and the general practitioner‐led Bridges pathway.^19^ Cultural competency in health care generally influences improved access and health outcomes in racial/ethnic minority groups in the community by increasing awareness, knowledge, and skills of healthcare providers or patients as well as modifying policies and practices of organizations.^35^ Organizational cultural competency such as the use of bilingual community health workers, interpreters, and patient navigators has been found to improve access in health service‐based settings. At the health provider level, some of the interventions include training, workshops to improve the understanding of cultural beliefs in the community, and interpersonal skills for delivering culturally‐sensitive care.^35^, ^36^ Culturally‐appropriate framework with strategies, for example, respectful, trustworthy communications and time for genuine engagement with patients, are considered important to support women in expressing their menstrual symptoms and make them feel comfortable with gynecologic examinations.^37^ Furthermore, multicultural interventions have the potential to improve access to patients from different cultural backgrounds that can result in equitable health outcomes.^35^

Similarly, we did not find any study targeting approachability or the existence of reachable services, meaning services that overcome geographic and time barriers. With women having social roles such as work and family commitments that are prioritized over attending clinical appointments for AUB,^4^ interventions focusing on locally accessible gynecologic care without the need for travel to hospitals could be valuable. An outreach women's clinic targeting rural communities, communities with ethnic minority groups, and low‐resource settings has been shown to improve the uptake of cervical screening^38^ and could be valuable in the context of providing a reachable service for women with AUB.

Patient‐level interventions in this review focus on the use of patient information and decision tools to improve the ability to engage and shared decision making in consultations. This resonates with a previous systematic review on interventions supporting shared decision making for women with HMB,^39^ which found decision aids to be helpful but could be improved with more attention to the collaborative element. Interventions using computer‐ or web‐based decision aids conducted in western and high‐income countries could result in generalizability issues with replicating the study in countries with limited access to technology.

This review identified lack of studies at an individual or community level targeting ability to perceive (health literacy and beliefs), ability to seek (personal and social values, culture), ability to reach (transport, mobility, living environment, social support), and ability to pay (income, health insurance). A recent Cochrane review^40^ found no health education interventions to promote access, including early presentation and early referral, for women with abnormal uterine bleeding. Culturally‐appropriate health education that is tailored to the cultural and religious beliefs and linguistics skills of the community have been found to be useful. A systematic review of interventions to increase the uptake of cervical screening in lower socio‐economic settings identified that health education and self‐testing have improved cervical screening in the community.^38^ Educational interventions could be helpful for improving awareness of the significance of symptoms of AUB among women. A review identifying 50 different instruments used to evaluate menstrual symptoms and quality of life for AUB also found that there was wide variability in the use of instruments, and none was considered as a standard outcome measure.^41^ The instruments lacked evidence for feasibility and acceptability for use by women. A culturally‐tailored self‐screening tool to recognize the symptoms of AUB that addresses all psychometric properties and is easy and acceptable to use for women could be valuable. Interventions targeting the ability to perceive, seek, and reach have the potential to decrease the time between identification of symptoms and receiving diagnosis and treatment. Furthermore, interventions targeting reach and payment for the services would be helpful for women with variable socio‐economic needs.

A strength of this study is that we used a broad range of search terms for AUB and healthcare access in our search strategy. The database search was conducted from inception until the recent date for the current review. A limitation is that we did not search gray literature and studies published in languages other than English.

In conclusion, interventions to improve access to women with abnormal uterine bleeding are restricted to organizational interventions targeting mainly the availability and affordability of services in a hospital setting. Future research should aim at interventions in the primary care setting and community setting. Interventions with a focus on approachability, acceptability, appropriateness of services, and ability to perceive, seek, reach, and pay for services is required. Interventions targeting education for general practitioners, culturally‐tailored information resources, and a culturally‐safe and supported environment could improve access to health care for women with different cultural backgrounds and improve equitable health outcomes.

## CONFLICTS OF INTEREST

The authors have no conflicts of interests.

## AUTHOR CONTRIBUTIONS

All the authors have contributed to the review design. PSK and SF were involved in the screening and selection of articles. PSK led the manuscript writing. All authors contributed to the review of the manuscript and approval of the final draft of the manuscript.

## Supporting information


Appendix S1

Appendix S2

## Data Availability

Data available in article supplementary file
