# Supplementary material for: Interventions to improve access to care for abnormal uterine bleeding: A systematic scoping review
Source: Int J Gynaecol Obstet. 2022 May 5;160(1):38–48. doi: 10.1002/ijgo.14224 (PMC10084285; doi:10.1002/ijgo.14224)

**Appendix A: Search strategy**

**EMBASE - All years <1947-Present with Daily Update>**

| Search history sorted by search number ascending | | | | |  |  |  |  |
| --- | --- | --- | --- | --- | --- | --- | --- | --- |
| **#** | **Searches** | **Results** | | **Type** | |  |  |  |
|  | | | | | | | |  |
| 1 | *menstruation disorder/ or *"amenorrhea and oligomenorrhea"/ or *amenorrhea/ or *menstrual irregularity/ or *oligomenorrhea/ or *"menorrhagia and metrorrhagia"/ or *menorrhagia/ or *metrorrhagia/ | 10003 | | Advanced | |  |  |  |
| 2 | (uterine h?emorrhage* or abnormal uterine bleeding or AUB or heavy uterine bleeding or dysfunctional uterus or irregular menstrual bleeding or menstrual disorder or menstrual symptoms or irregular menstrual cycle* or postmenopausal bleeding or post-menopausal bleeding or menometrorrhagia or menorrhagia or heavy menstrual bleeding or heavy period* or hypermenorrhea or metrorrhagia or bleeding between periods or bleeding between cycles or breakthrough bleeding or break-through bleeding or dysfunctional uterine bleeding* or intermenstrual bleeding or inter-menstrual bleeding or menstrua* disturbance* or menstrua* disorder* or hypomenorrhea or irregular menses or irregular menstruation or menstrual irregularit* or polymenorrh* or amenorrh* or oligomenorrh* or prolonged menstrual bleeding or hyper-menorrhea or hypo-menorrhea or poly menorrhea or oligo menorrhea or oligo amenorrhea).ti,kw. | 12562 | | Advanced | |  |  |  |
| 3 | 1 or 2 | 16119 | | Advanced | |  |  |  |
| 4 | "health care cost"/ or "cost of illness"/ or health care access/ or health care delivery/ or right to health/ or integrated health care system/ or health equity/ or health care disparity/ or transcultural care/ or cultural nursing/ or indigenous health care/ or doctor patient relationship/ or health literacy/ or taboo/ or social belief/ or cultural anthropology/ or patient education/ or social stigma/ or attitude to health/ or health promotion/ or social psychology/ or empowerment/ or stigma/ or personal autonomy/ or relational autonomy/ or decision making/ or patient decision making/ or shared decision making/ or trust/ or motivation/ or environment/ or social environment/ or social status/ or income group/ or socioeconomics/ or highest income group/ or middle income group/ or lowest income group/ or working poor/ or social status/ or neet status/ or poverty/ or social background/ or social class/ or social stratification/ or community care/ or community health nursing/ or social support/ or psychosocial care/ or social isolation/ or social alienation/ or loneliness/ or social exclusion/ or ostracism/ or social norm/ or socialization/ or economic status/ or household economic status/ or "traffic and transport"/ or employment status/ or neet status/ or unemployment/ or employment/ or full time employment/ or parttime employment/ or permanent employment/ or self employment/ or supported employment/ or temporary employment/ or household income/ or income/ or family income/ or personal income/ or health insurance/ or blue cross blue shield/ or community-based health insurance/ or medicaid/ or medicare/ or national health insurance/ or private health insurance/ or public health insurance/ or universal health insurance/ or patient compliance/ or patient attitude/ or patient compliance/ or patient attitude/ or caregiver/ or medical geography/ or geography/ or geographic distribution/ or health personnel attitude/ or nurse attitude/ or physician assistant attitude/ or physician attitude/ or protocol compliance/ or hospital management/ or hospital admission/ or patient care/ or integrated health care system/ or health care quality/ or trust/ or motivation/ or professionalism/ or patient referral/ | 3046503 | | Advanced | |  |  |  |
| 5 | (access or accessibility or (service* adj2 utili#ation) or affordab* or appropriateness or approachab* or equit* or inequit* or equalit* or inequalit* or barrier* or facilitat* or referral* or (wait* adj2 time*) or (present* adj2 (time* or delay*)) or doctor-patient or physician-patient or clinician-patient or (provider adj3 (communicat* or relationship)) or taboo* or stigma* or ((AUB or abnormal uterine bleeding) adj1 aware*) or (health adj1 (literacy or education or knowledge or practices or promotion* or beliefs or insurance)) or attitude* or (patient* adj2 educat*) or personal values or social values or trust* or expectation* or culture* or gender* or autonomy or (decision adj2 (making or aid* or tool*)) or living environment or social support or transport* or income or empowerment or adherence or compliance or support or geograph* or professional values or screening or (hours adj2 open*) or appointment* or cost* or socio* or economic or poverty or employ* or coordination or co-ordination or continuity or care pathway or (integrated adj2 (care or healthcare or delivery)) or ((healthcare or health care) adj2 quality) or ((adequa* or availab*) adj5 (lack* or diagnos* or care* or resourc* or treat* or therap* or manage* or service*)) or followup or follow-up).ti,ab,kw. | 9542489 | | Advanced | |  |  |  |
| 6 | 4 or 5 | 10950289 | | Advanced | |  |  |  |
| 7 | 3 and 6 | 4255 | | Advanced | |  |  |  |
| 8 | postpartum amenorrhea.ti,kw. | 40 | | Advanced | |  |  |  |
| 9 | 7 not 8 | 4239 | | Advanced | |  |  |  |
| 10 | limit 9 to english language | 3883 | | Advanced | |  |  |  |
| 11 | limit 10 to conference abstracts | 646 | | Advanced | |  |  |  |
| 12 | 10 not 11 | 3237 | | Advanced | |  |  |  |
|  | | |  | |  |  |  |  |

**Ovid MEDLINE(R) ALL <1946 to February 26, 2021>**

| Search history sorted by search number ascending | | | |  |  |  |
| --- | --- | --- | --- | --- | --- | --- |
| **#** | **Searches** | **Results** | **Type** |  |  |  |
|  | | | | | | |
| 1 | *menorrhagia/ or *metrorrhagia/ or *menstruation disturbances/ or *amenorrhea/ or *oligomenorrhea/ or *uterine h?emorrhage/ | 18723 | Advanced |  |  |  |
| 2 | (uterine h?emorrhage or abnormal uterine bleeding or AUB or heavy uterine bleeding or dysfunctional uterus or irregular menstrual bleeding or menstrual disorder or menstrual symptoms or irregular menstrual cycle* or postmenopausal bleeding or post-menopausal bleeding or menometrorrhagia or menorrhagia or heavy menstrual bleeding or heavy period* or hypermenorrhea or metrorrhagia or bleeding between periods or bleeding between cycles or breakthrough bleeding or break-through bleeding or dysfunctional uterine bleeding* or intermenstrual bleeding or inter-menstrual bleeding or menstrua* disturbance* or menstrua* disorder* or hypomenorrhea or irregular menses or irregular menstruation or menstrual irregularit* or polymenorrh* or amenorrh* or oligomenorrh* or prolonged menstrual bleeding or hyper-menorrhea or hypo-menorrhea or poly menorrhea or oligo menorrhea or oligo amenorrhea).ti,kw,kf. | 13410 | Advanced |  |  |  |
| 3 | uterine hemorrhage/ or menorrhagia/ or metrorrhagia/ or menstruation disturbances/ or amenorrhea/ or menorrhagia/ or oligomenorrhea/ | 30851 | Advanced |  |  |  |
| 4 | (uterine h?emorrhage or abnormal uterine bleeding or AUB or heavy uterine bleeding or dysfunctional uterus or irregular menstrual bleeding or menstrual disorder or menstrual symptoms or irregular menstrual cycle* or postmenopausal bleeding or post-menopausal bleeding or menometrorrhagia or menorrhagia or heavy menstrual bleeding or heavy period* or hypermenorrhea or metrorrhagia or bleeding between periods or bleeding between cycles or breakthrough bleeding or break-through bleeding or dysfunctional uterine bleeding* or intermenstrual bleeding or inter-menstrual bleeding or menstrua* disturbance* or menstrua* disorder* or hypomenorrhea or irregular menses or irregular menstruation or menstrual irregularit* or polymenorrh* or amenorrh* or oligomenorrh* or prolonged menstrual bleeding or hyper-menorrhea or hypo-menorrhea or poly menorrhea or oligo menorrhea or oligo amenorrhea).ti,ab. | 29797 | Advanced |  |  |  |
| 5 | 3 and 4 | 13991 | Advanced |  |  |  |
| 6 | 1 or 2 or 5 | 24624 | Advanced |  |  |  |
| 7 | Health Expenditures/ or "cost of illness"/ or health services accessibility/ or health equity/ or healthcare disparities/ or Culturally Competent Care/ or Physician-Patient Relations/ or Health Literacy/ or Taboo/ or Culture/ or Patient Education as Topic/ or social stigma/ or Health Knowledge, Attitudes, Practice/ or Health Promotion/ or Social Values/ or Personal Autonomy/ or Relational Autonomy/ or Decision Making/ or Trust/ or Motivation/ or Environment/ or social conditions/ or social environment/ or community networks/ or social support/ or psychosocial support systems/ or social isolation/ or loneliness/ or social alienation/ or social marginalization/ or social norms/ or socialization/ or socioeconomic factors/ or economic status/ or poverty/ or poverty areas/ or social class/ or Transportation/ or employment/ or income/ or Insurance, Health/ or empowerment/ or Patient Compliance/ or attitude to health/ or "patient acceptance of health care"/ or Caregivers/ or Geography/ or "Attitude of Health Personnel"/ or "Attitude of Health Personnel"/ or "appointments and schedules"/ or waiting lists/ or "Continuity of Patient Care"/ or "Delivery of Health Care, Integrated"/ or "Quality of Health Care"/ or trust/ or motivation/ or professionalism/ or "Referral and Consultation"/ or Delivery of Healthcare/ | 1528544 | Advanced |  |  |  |
| 8 | (access or accessibility or (service* adj2 utili#ation) or affordab* or appropriateness or approachab* or equit* or inequit* or equalit* or inequalit* or barrier* or facilitat* or referral* or (wait* adj2 time*) or (present* adj2 (time* or delay*)) or doctor-patient or physician-patient or clinician-patient or (provider adj3 (communicat* or relationship)) or taboo* or stigma* or ((AUB or abnormal uterine bleeding) adj1 aware*) or (health adj1 (literacy or education or knowledge or practices or promotion* or beliefs or insurance)) or attitude* or (patient* adj2 educat*) or personal values or social values or trust* or expectation* or culture* or gender* or autonomy or (decision adj2 (making or aid* or tool*)) or living environment or social support or transport* or income or empowerment or adherence or compliance or support or geograph* or professional values or screening or (hours adj2 open*) or appointment* or cost* or socio* or economic or poverty or employ* or coordination or co-ordination or continuity or care pathway or (integrated adj2 (care or healthcare or delivery)) or ((healthcare or health care) adj2 quality) or ((adequa* or availab*) adj5 (lack* or diagnos* or care* or resourc* or treat* or therap* or manage* or service*)) or followup or follow-up).ti,ab,kw,kf. | 7242533 | Advanced |  |  |  |
| 9 | 7 or 8 | 7931287 | Advanced |  |  |  |
| 10 | 6 and 9 | 4567 | Advanced |  |  |  |
| 11 | Postpartum amenorrhea.ti,kw. | 206 | Advanced |  |  |  |
| 12 | 10 not 11 | 4466 | Advanced |  |  |  |
| 13 | limit 12 to english language | 4046 | Advanced |  |  |  |
|  | | |  |  |  |  |

**Scopus**

( ( ( TITLE ( ( "uterine h*emorrhage*" OR "abnormal uterine bleeding" OR aub OR "heavy uterine bleeding" OR "dysfunctional uterus" OR "irregular menstrual bleeding" OR "menstrual disorder" OR "menstrual symptoms" OR "irregular menstrual cycle*" OR "postmenopausal bleeding" OR "post-menopausal bleeding" OR menometrorrhagia OR menorrhagia OR "heavy menstrual bleeding" OR "heavy period*" OR hypermenorrhea OR metrorrhagia OR "bleeding between periods" OR "bleeding between cycles" OR "breakthrough bleeding" OR "break-through bleeding" OR "dysfunctional uterine bleeding*" OR "intermenstrual bleeding" OR "inter-menstrual bleeding" OR "menstruation disturbance*" OR "menstrual disturbance*" OR "menstruation disorder*" OR hypomenorrhea OR "irregular menses" OR "irregular menstruation" OR "menstrual irregularit*" OR polymenorrh* OR amenorrh* OR oligomenorrh* OR "prolonged menstrual bleeding" OR hyper-menorrhea OR hypo-menorrhea OR poly-menorrhea OR oligo-menorrhea OR oligo-amenorrhea ) ) ) OR ( AUTHKEY ( ( "uterine h*emorrhage*" OR "abnormal uterine bleeding" OR aub OR "heavy uterine bleeding" OR "dysfunctional uterus" OR "irregular menstrual bleeding" OR "menstrual disorder" OR "menstrual symptoms" OR "irregular menstrual cycle*" OR "postmenopausal bleeding" OR "post-menopausal bleeding" OR menometrorrhagia OR menorrhagia OR "heavy menstrual bleeding" OR "heavy period*" OR hypermenorrhea OR metrorrhagia OR "bleeding between periods" OR "bleeding between cycles" OR "breakthrough bleeding" OR "break-through bleeding" OR "dysfunctional uterine bleeding*" OR "intermenstrual bleeding" OR "inter-menstrual bleeding" OR "menstruation disturbance*" OR "menstrual disturbance*" OR "menstruation disorder*" OR hypomenorrhea OR "irregular menses" OR "irregular menstruation" OR "menstrual irregularit*" OR polymenorrh* OR amenorrh* OR oligomenorrh* OR "prolonged menstrual bleeding" OR hyper-menorrhea OR hypo-menorrhea OR poly-menorrhea OR oligo-menorrhea OR oligo-amenorrhea ) ) ) ) AND ( TITLE-ABS-KEY ( ( ( access OR accessibility OR ( service* W/1 utili?ation ) OR affordab* OR appropriateness OR approachab* OR equit* OR inequit* OR equalit* OR inequalit* OR barrier* OR facilitat* OR referral* OR ( wait* W/1 time* ) OR ( present* W/1 ( time* OR delay* ) ) OR doctor-patient OR physician-patient OR clinician-patient OR ( provider W/2 ( communicat* OR relationship ) ) OR taboo* OR stigma* ) ) OR ( health W/0 ( literacy OR education OR knowledge OR practices OR promotion* OR beliefs OR insurance ) ) OR attitude* OR ( patient* W/1 educat* ) OR "personal values" OR "social values" OR trust* OR expectation* OR culture* OR gender* OR autonomy OR ( decision W/1 ( making OR aid* OR tool* ) ) OR "living environment" OR "social support" OR transport* OR income OR empowerment OR adherence OR compliance OR support OR geograph* OR "professional values" OR screening OR ( hours W/1 open* ) OR appointment* OR cost* OR socio* OR economic OR poverty OR employ* OR coordination OR co-ordination OR continuity OR "care pathway" OR ( integrated W/1 ( care OR healthcare OR delivery ) ) OR ( ( quality W/1 ( healthcare OR "health care" ) ) OR ( ( adequa* OR availab* ) W/4 ( lack* OR diagnos* OR care* OR resourc* OR treat* OR therap* OR manage* OR service* ) ) OR followup OR follow-up ) ) ) ) AND NOT ( TITLE ( "postpartum amenorrhea" ) ) AND ( LIMIT-TO ( LANGUAGE , "English" ) )

**EBM Reviews - Cochrane Central Register of Controlled Trials**

| Search history sorted by search number ascending | | | | |  |  |  |
| --- | --- | --- | --- | --- | --- | --- | --- |
| **#** | **Searches** | **Results** | **Type** |  |  |  |  |
|  | | | | | | | |
| 1 | *menorrhagia/ or *metrorrhagia/ or *menstruation disturbances/ or *amenorrhea/ or *oligomenorrhea/ or *uterine h?emorrhage/ | 3 | Advanced |  |  |  |  |
| 2 | (uterine h?emorrhage or abnormal uterine bleeding or AUB or heavy uterine bleeding or dysfunctional uterus or irregular menstrual bleeding or menstrual disorder or menstrual symptoms or irregular menstrual cycle* or postmenopausal bleeding or post-menopausal bleeding or menometrorrhagia or menorrhagia or heavy menstrual bleeding or heavy period* or hypermenorrhea or metrorrhagia or bleeding between periods or bleeding between cycles or breakthrough bleeding or break-through bleeding or dysfunctional uterine bleeding* or intermenstrual bleeding or inter-menstrual bleeding or menstrua* disturbance* or menstrua* disorder* or hypomenorrhea or irregular menses or irregular menstruation or menstrual irregularit* or polymenorrh* or amenorrh* or oligomenorrh* or prolonged menstrual bleeding or hyper-menorrhea or hypo-menorrhea or poly menorrhea or oligo menorrhea or oligo amenorrhea).ti,hw. | 3679 | Advanced |  |  |  |  |
| 3 | uterine hemorrhage/ or menorrhagia/ or metrorrhagia/ or menstruation disturbances/ or amenorrhea/ or menorrhagia/ or oligomenorrhea/ | 1597 | Advanced |  |  |  |  |
| 4 | (uterine h?emorrhage or abnormal uterine bleeding or AUB or heavy uterine bleeding or dysfunctional uterus or irregular menstrual bleeding or menstrual disorder or menstrual symptoms or irregular menstrual cycle* or postmenopausal bleeding or post-menopausal bleeding or menometrorrhagia or menorrhagia or heavy menstrual bleeding or heavy period* or hypermenorrhea or metrorrhagia or bleeding between periods or bleeding between cycles or breakthrough bleeding or break-through bleeding or dysfunctional uterine bleeding* or intermenstrual bleeding or inter-menstrual bleeding or menstrua* disturbance* or menstrua* disorder* or hypomenorrhea or irregular menses or irregular menstruation or menstrual irregularit* or polymenorrh* or amenorrh* or oligomenorrh* or prolonged menstrual bleeding or hyper-menorrhea or hypo-menorrhea or poly menorrhea or oligo menorrhea or oligo amenorrhea).ti,ab. | 4701 | Advanced |  |  |  |  |
| 5 | 3 and 4 | 894 | Advanced |  |  |  |  |
| 6 | 1 or 2 or 5 | 3679 | Advanced |  |  |  |  |
| 7 | Health Expenditures/ or "cost of illness"/ or health services accessibility/ or health equity/ or healthcare disparities/ or Culturally Competent Care/ or Physician-Patient Relations/ or Health Literacy/ or Taboo/ or Culture/ or Patient Education as Topic/ or social stigma/ or Health Knowledge, Attitudes, Practice/ or Health Promotion/ or Social Values/ or Personal Autonomy/ or Relational Autonomy/ or Decision Making/ or Trust/ or Motivation/ or Environment/ or social conditions/ or social environment/ or community networks/ or social support/ or psychosocial support systems/ or social isolation/ or loneliness/ or social alienation/ or social marginalization/ or social norms/ or socialization/ or socioeconomic factors/ or economic status/ or poverty/ or poverty areas/ or social class/ or Transportation/ or employment/ or income/ or Insurance, Health/ or empowerment/ or Patient Compliance/ or attitude to health/ or "patient acceptance of health care"/ or Caregivers/ or Geography/ or "Attitude of Health Personnel"/ or "Attitude of Health Personnel"/ or "appointments and schedules"/ or waiting lists/ or "Continuity of Patient Care"/ or "Delivery of Health Care, Integrated"/ or "Quality of Health Care"/ or trust/ or motivation/ or professionalism/ or "Referral and Consultation"/ or Delivery of Healthcare/ | 51414 | Advanced |  |  |  |  |
| 8 | (access or accessibility or (service* adj2 utili#ation) or affordab* or appropriateness or approachab* or equit* or inequit* or equalit* or inequalit* or barrier* or facilitat* or referral* or (wait* adj2 time*) or (present* adj2 (time* or delay*)) or doctor-patient or physician-patient or clinician-patient or (provider adj3 (communicat* or relationship)) or taboo* or stigma* or ((AUB or abnormal uterine bleeding) adj1 aware*) or (health adj1 (literacy or education or knowledge or practices or promotion* or beliefs or insurance)) or attitude* or (patient* adj2 educat*) or personal values or social values or trust* or expectation* or culture* or gender* or autonomy or (decision adj2 (making or aid* or tool*)) or living environment or social support or transport* or income or empowerment or adherence or compliance or support or geograph* or professional values or screening or (hours adj2 open*) or appointment* or cost* or socio* or economic or poverty or employ* or coordination or co-ordination or continuity or care pathway or (integrated adj2 (care or healthcare or delivery)) or ((healthcare or health care) adj2 quality) or ((adequa* or availab*) adj5 (lack* or diagnos* or care* or resourc* or treat* or therap* or manage* or service*)) or followup or follow-up).ti,ab,hw. | 642254 | Advanced |  |  |  |  |
| 9 | 7 or 8 | 645067 | Advanced |  |  |  |  |
| 10 | 6 and 9 | 1404 | Advanced |  |  |  |  |
| 11 | Postpartum amenorrhea.ti. | 0 | Advanced |  |  |  |  |
| 12 | 10 not 11 | 1404 | Advanced |  |  |  |  |
| 13 | limit 12 to english language | 1218 | Advanced |  |  |  |  |
| CINAHL | | |  | |  |  |  |


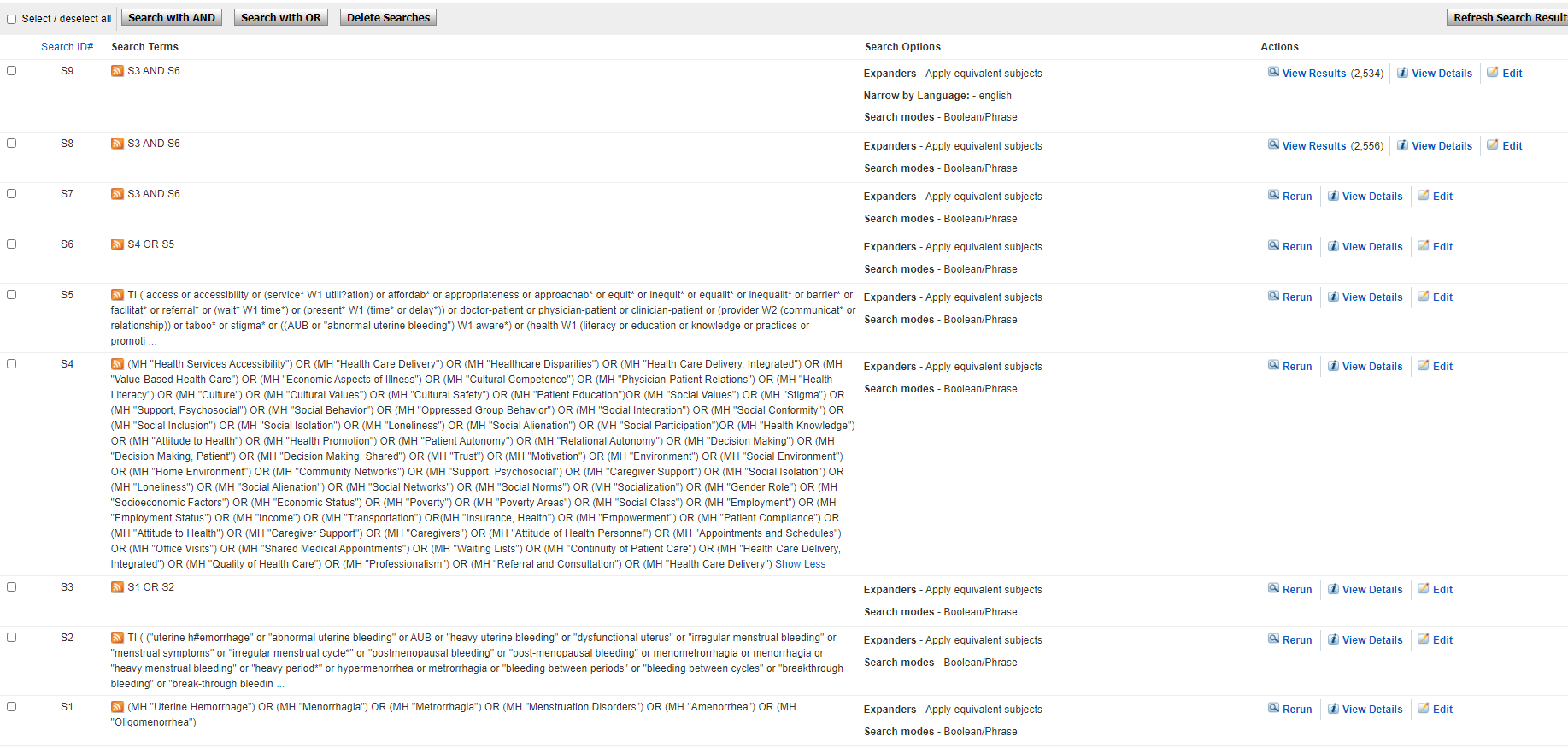

Supplement: Supplementary file 1 — Appendix S1 [file IJGO-160-38-s002.docx]
