# Supplementary material for: Interventions to improve access to care for abnormal uterine bleeding: A systematic scoping review
Source: Int J Gynaecol Obstet. 2022 May 5;160(1):38–48. doi: 10.1002/ijgo.14224 (PMC10084285; doi:10.1002/ijgo.14224)
Supplement: Supplementary file 2 — Appendix S2 [file IJGO-160-38-s001.docx]

| **Data extraction- Interventions to improve access to care for AUB** | | | | | | | |
| --- | --- | --- | --- | --- | --- | --- | --- |
| **Author/yr**  **Country** | **Study type** | **Population/ Setting** | **Sample size** | **Intervention** | **Dimension of access/outcome measures** | **Instrument** | **Findings** |
| **Organisational interventions (Creating multidisciplinary team)** | | | | | | | |
| Wygant 2019 (12)  Minnesota, USA | Quasi experimental  Before-after study  (*The hysteroscopy wait time data were compared from the*  *preintervention time period of January to June 2015 to the*  *postintervention time period of January to June 2016)* | Condition: AUB  Age: Not available  Setting: academic centre (Mayo clinic) - outpatient gynaecology service | Pre int-393  Post int-647 | Collaborative hysteroscopy clinic  (collaborative women’s health nurse practitioner and gynaecologist practice) | Availability  (Waiting time for appointment,  Number of appointment slots) | Patient medical records | The appointment slots available were increased from 423 to approximately 735 in the intervention period  (an increase of 57.5%).  63% of patients were scheduled within 0–13 days of initial contact, compared with 30% of patients before intervention period. |
| **Organisational interventions (Bringing services together)** | | | | | | | |
| Lotfallah 2005 (13)  United Kingdom | Retrospective study  *(To evaluate the role and feasibility of 1 stop clinic, a retrospective analysis of 308 patients referred to the clinic)* | Condition: PMB  Age (mean): 59.7 years  (range, 42-91)  Setting: General Hospital  (Barnsley district general hospital) | N= 308 | One stop clinic  (transabdominal and transvaginal ultrasound scan with hysteroscopy | Availability  (Number of patients managed in first visit) | Retrospective record analysis | A total of 216 of 308 (70%) patients were totally managed and discharged at the first visit with no further follow-up, and hospital admission was avoided in 258 (83.7%) of cases.  Conclusion: The 1-stop clinic is effective in reducing the number of hospital visits per patient as well as hospital admissions and the waiting list. |
| Sulaiman 2004 (14)  Glasgow, UK | Retrospective study  *(Retrospective studying running from January to July 2003 comparing the one-stop clinic with four traditional consultant led outpatient gynaecology*  *clinics also seeing women with PMB running concurrently in the same hospital.)* | Condition: PMB  Age: 59.0 (57.3 - 60.8) Vs 58.7 (56.0 - 61.4)  Setting: General  Hospital  *(Glasgow city hospital)* | One stop clinic -95  General clinic -51 | One stop post menopausal clinic  (gynaecological history, abdominal palpation TVS and endometrial sampling)  Vs  General gynaecology clinic | Availability  (Waiting times, number of visits to hospital, time from first consultation to a definitive management plan or discharge letter). | Retrospective chart review | Time from referral to first consultation was shorter in the PMB clinic (35 ± 17 days, p<O.OOI) and the women had fewer visits (1.3 ±O,6 visits, P < 0.00 I).  The time from first consultation to a definitive management plan was shorter in the PMB clinic (6±6 days, p<O.OOI). Sixty-eight women (72%) in the PMB clinic received immediate reassurance and were discharged after the first consultation compared to six per cent from the general clinic. |
| Mohammad 2003 (15)  Durham, UK | Prospective study  (*We analysed the prospective findings of 80 patients who met the criteria of direct GP referral to PMB clinic.)* | Condition: PMB  Age(mean,range): 58, (45-84) yrs  Setting: General Hospital outpatient  (*Bishop Auckland General Hospital,*  *Durham*) | N= 80 | One stop PMB clinic  (consultation, transvaginal ultrasound (TVS) and pipelle endometrial sampling in one visit) | Availability  (Referral time, waiting time, number of patients managed in single visit)  Appropriateness  (satisfaction with service) | Patient records  (findings were recorded prospectively on a designed form.) | The mean time from  GP referral to consultation at PMB clinic was 14.8 days (range:2–24 days).  89% of the patients were managed on the basis of a single visit. Only nine patients (11%) required further visits to the clinic to discuss histology results and rescanning for reassurance.  Seventy women (87.5%) considered a single PMB clinic visit was satisfactory. |
| Abu 2001 (16)  United Kingdom | Prospective study  *(the women included in this prospective study were recruited from those who attended the one-stop menstrual clinic and those with similar complaints who attended the traditional gynaecology clinic)* | Condition: Menstrual disorder  Age: 42.3 (34-54.8) Vs 42.1 (35.3-54)  Setting: General Hospital outpatient | Survey  Gynae clinic 106 Vs OSMC 98)  Interviews Gynae clinic 13 Vs OSMC 22 | Gynaecology clinic  Vs  One stop menstrual clinic (completion of all investigations in one day-haematology, pelvic ultrasound, hysteroscopy and endometrial biopsy, provison of information to patients) | Availability  (waiting for appointment)  Appropriateness (coordination, continuity and clinic organisation)  Ability to engage (provision of information to patients)  Ability to seek  (choice of doctor) | Patient career diary questionnaire  Qualitative interviews | Significant positive experience with one stop menstrual clinic on all domains  Qualitative interview confirmed one stop clinic was more suited to the needs of patients |
| Dueholm 1999 (17)  Denmark | Prospective study  (*to evaluate a one-stop outpatient bleeding*  *disorder clinic, review of investigations and management of patients attending the clinic)* | Condition: AUB with bleeding disorder  Age: 44.4 (22–59)  Setting: General Hospital outpatient bleeding disorder clinic) | N=106 | One stop clinic (with pelvic ultrasound, hysterosonography and,  when indicated, endometrial sampling) | Availability  (number of patients receiving diagnosis and treatment plan in first visit) | Not reported | In 93% of the patients a sufficient diagnosis of the uterine cavity was attained at a one-stop visit. In 73% of the patients a treatment plan could be formulated during the consultation. |
| Atiomo 1998 (18)  United Kingdom | Retrospective study  *(to evaluate the investigation*  *of post-menopausal bleeding at this clinic, the records of 212 women*  *seen in this clinic in 1994 were reviewed)* | Condition: PMB  Age(mean): 62 years  (range 43-91 years)  Setting: General Hospital  (Derriford hospital, Plymouth, UK) | N=212 | One stop clinic  (with gynaecological examination, transvaginal ultrasound and endometrial biopsy) | Availability  (waiting time between referral by the general practitioner  and clinic appointment,  number of visits to the gynaecology department required to make a diagnosis or provide reassurance) | Review of records | One hundred and forty-four women (67.9%) were evaluated and reassured in one visit.  **Reduced number of visits for diagnosis**.  Mean waiting period after GP referral was 36 days (range 6-157 days) |
| **Organisational interventions (Continuity of care)** | | | | | | | |
| Julian 2007 (19)  United Kingdom | Prospective  study  *A prospective, non-random comparison of two services: women attending the new (Bridges) pathway*  *compared with those attending a consultant-led one-stop menstrual clinic (OSMC)* | Condition: HMB  Age: Not available  Setting: Teaching hospital and general practices with one primary care trust | Bridges 99  Vs  Consultant led clinic 94 | GP led Bridges pathway  **Vs**  Consultant–led One Stop Menstrual Clinic (OSMC) | Availability  (Waiting time, fitting with appointment, number of outpatient appointments)  Ability to seek  (choice of doctor and limbo-patient experience of non-coordination between primary and secondary care) | Patient carrier diary | Bridges improved patient information, fitting in with appointments (ease of access), choice of doctor, waiting time, and less ‘‘limbo’’ (patient experience of non-coordination between primary  and secondary care)  -Fewer outpatient appointments in Bridges group |
| **Organisational interventions (procedure in outpatient setting)** | | | | | | | |
| Bennett 2020 (20)  Canada | Retrospective study with probabilistic decision tree model | Condition: AUB  Age: 45.3 ± 12.2  Setting: Women’s hospital (The Ottawa Hospital’s Shirley E. Greenberg Women’s  Health Centre) | 200 (randomly selected) | outpatient uterine assessment and treatment unit  (UATU)  Vs  Usual care | Affordability  (total cost of women)  Availability  (time savings per patient) | Retrospective chart review  (between Apr. 1, 2014, and Mar. 31, 2017) | Compared with usual care, care in the UATU was associated with a decrease in overall cost ($1332, 95% confidence interval  [CI] –$1742 to –$1008) and a decrease in overall time to treatment |
| Diwakar 2016 (21)  United Kingdom | RCT  (*The OPT trial is the first large randomised prospective*  *Study*) | Condition: AUB  Age: Not available  Setting: Thirty-one secondary care UK NHS hospitals | Outpatient 254  Inpatient 253 | outpatient polyp treatment (OPT)  Vs  inpatient polyp treatment | Affordability  (cost utility of treatment) | patient self-assessment  questionnaires on quality adjusted life years (QALYs)  -Treatment costs  -Out of pocket costs. | Outpatient treatment of uterine polyps associated  with abnormal uterine bleeding appears to be more cost-effective  than inpatient treatment at willingness-to-pay thresholds  acceptable to the NHS |
| Moawad 2014 (22)  Florida, USA | Retrospective study | Condition: AUB  Age: 47 (10)  Setting: 2 outpatient clinics in an academic university Setting (University of Florida women’s health centre) | N=130 | Office hysteroscopy  Vs  Hysteroscopy in operating room (OR) | Affordability  (cost of treatment) | paper charts and electronic medical records | Conducting office hysteroscopy and then referring the patient for hysteroscopy in the OR only if needed resulted in more cost savings when compared to operating room hysteroscopy only. |
| Ahonkallio 2012 (23)  Finland | Retrospective study  (The strength of this study is that it is based on real reported resource use, not a model) | Condition: HMB  Age: Not available  Setting: Teaching general hospital (Oulu University  Hospital) | outpatient-16  Daycase- 20 | outpatient endometrial ablation  Vs  inpatient endometrial ablation | Affordability  (cost of a day case procedure performed under general anaesthesia in the  operating theatre and an outpatient procedure performed using local anaesthesia) | Hospital’s operative database and information from financial planning department | The outpatient procedure  was 800 euros cheaper than the daycase procedure for the  health service provider. The difference is caused by lower costs of  the hospital ward and anaesthesia, and partly by overhead costs. |
| Jack 2005 (24)  United kingdom | RCT | Condition: HMB  Age: Outpatient- 42.36 [4.78]; Daycase - 42.41 [5.35]  Setting: A large teaching hospital | Outpatient-97  OT-100 | Outpatient microwave endometrial ablation (MEA)  Vs  standard MEA treatment in a daycase theatre with hormonal preparation | Affordability  (cost of treatment) | Health service costs  Costing questionnaires for non-health service costs (e.g. transport)  . | The mean health service costs were £124 (95%  CI £86–194) lower for the patients in the post-menses group (out patient MEA). |
| **Patient education** | | | | | | | |
| Aarts 2021 (25)  USA | Quasi experimental before-after study  *(We adopted a before-and-after study design, comparing a preintervention*  *usual care phase (T1) to an intervention phase (T2))* | Condition: HMB  Age (mean,SD):  Pre int -38.5 (10.46)  Post int- 40.6 (5.37)  Setting: Outpatient gynaecology service in an academic medical centre and two community group practices | Pre intervention (n=28)  Post intervention (n=32) | Option grid encounter decision aid (Information on available treatment options and training to clinicians) | Ability to engage  *(shared decision making)* | Questionnaires  - Patient reported shared decision making (*CollaboRATE*)  -Decisional conflict (*SURE test*)  Audio recorded Consultation  - Observer reported shared decision making (*observer OPTION*) | Encounter decision aid during counselling resulted in greater levels of shared decision making both from a patient’s and observer’s perspective. |
| Dietrich 2017  (26)  Texas, USA | Prospective study  *(This was a*  *prospective cohort study)* | Condition: HMB with bleeding disorder  Age: 14.1 ± 1.9 years adolescents  Setting: Children’s Hospital  *(Young*  *Women's Bleeding Disorder Clinic (YWBDC) at Texas Children's*  *Hospital)* | N=23 | Patient education-(ipod touch device with iperiod application) | Ability to engage  (patient information, adherence to medicine) | Retrospective chart review and patient information completed in ITD  -Patient compliance to medication and hospital admission | Improved compliance and fewer hospital days related to use of the device.  As a result of missed medications, 19% (4/22) of control group enrollees were admitted to the hospital for 1-2 days compared to none admitted from intervention group.  Conclusion: ITD is an excellent tool for adolescents with HMB and BD to allow self-monitoring, provider monitoring, and improve  educational access through engaging technology |
| Hess 2015  (27)  USA | RCT  *(This randomized trial was designed to evaluate*  *a preference elicitation tool)* | Condition: AUB  Age: 41.2 (7.9) vs 40.8 (8.3)  Setting: Women’s Hospital  *(gynecology clinics (Southern Pines Women’s Health Center, Southern Pines, NC, USA;*  *Primary Care Clinic of Wishard Hospital, Cottage Corner Clinic, or Coleman Center for Women, Indianapolis, IN,*  *USA) were enrolled)* | Int group-183  Control grp- 191 | Preference elicitation tool using Adaptive conjoint analysis (ACA)  Vs  Usual counselling | Ability to engage *(shared decision making)* | survey questionnaires at 6 weeks  -Decision regret (Decision Regret Scale)  -Satisfaction with care  *(Functional Assessment of Chronic Illness Therapy-Treatment Satisfaction-Patient Scale (FACIT-*  *TS-PS))* | A preference elicitation tool at the initial consultation visit did not reduce decision regret or improve treatment satisfaction among patients with AUB |
| Protheroe 2007 (28)  United Kingdom | RCT with 6 month follow-up | Condition: HMB with bleeding disorder  Age: control 41 (±5.4) years  Intervention 41 (±5.2) years  Setting: Primary care (19 general practices) | Control n=72 Intervention n=74 | The Clinical Guidance Tree (self-directed interactive computerised decision aid)  **Vs** Patient information leaflet | Ability to engage *(shared decision making)* | Postal questionnaires  *Primary outcome*: Decisional Conflict Scale  *Secondary outcome*:  State-Trait Anxiety Inventory, Menorrhagia Specific Utility Scale, knowledge about menorrhagia self-report questionnaire | significantly less decisional conflict in the intervention  group. A computerized decision aid, used outside of the primary care consultation, is effective in increasing patient involvement in decision making in primary care. |
| Vuorma 2003; (29,30)  Finland | RCT and Pre-trial prospective study  *(Randomized trial and a pre-trial prospective cohort study***)** | Condition: HMB  Age (mean,SD):  Inte- 44.5 (0.31)  Control - 44.3 (0.31)  Pre-trial - 44.3 (0.29)  Setting: General hospital gynaecology outpatient clinic.  (*gynaecology outpatient*  *clinics of 14 hospitals (four university teaching*  *hospitals, five central and five local hospitals).* | N=363  Intervention= 184  Control=179  Cohort study=206 | Information booklet vs usual care | Ability to engage  (Patient information, communication with personnel)  Affordability  (cost of treatment)  Availability  number of patients with planned treatment | Questionnaires  -Knowledge of AUB  -Satisfaction with communication with personnel in clinc  -Anxiety level  Medical records for information on treatment plans and treatment  - | **No between-group differences** were detected  in the change in anxiety, satisfaction or knowledge level  There were no marked disparities in treatment costs between groups.  *Treatment decision within 3 months was made more often in the intervention group than in the control group (96% and 89%*  *respectively, P ¼ 0.02).* |
| Kennedy 2002 (31)  Southwest England, UK | RCT  *(a randomised controlled study with 2 years of follow up.* | Condition: HMB  Age (mean,SD):  Control-40 (7.0)  Info – 40 (7.2)  Interview- 41 (6.9)  Setting: 6 general hospitals | Control- 298  Intervention:  Info-296  Interview-300 | control group- standard practice Information group-booklet and videotape before 6 weeks of consultation  Interview group –Interview (preference elicitation) along with information. | Ability to engage  (shared decision making)  Affordability  (cost of treatment) | Questionnaires (satisfaction rating)  At baseline, 6, 12 and 24 months | Interview group reported significantly higher opportunity for treatment decision making than control group.  Both intervention groups showed major mean cost savings in comparison with the control group. The interview group also showed cost savings when compared to information group. |
| **Community awareness** | | | | | | | |
| Hossenbaccus  2021 (32)  Canada | Quasi experimental before after Pilot study | Condition: HMB with bleeding disorder  Age: Grade 9 Girls  Setting: School  outreach programme  (11 different health classes at 3 high schools.) | Pre-intervention (n=163) post intervention (n-161) | Lets talk period(LTP) 75minutes class presentation-awareness of bleeding disorder symptoms | Ability to perceive  (Knowledge of menorrhagia and bleeding disorder) | Kahoot quizzies (online questionnaire) at baseline, post intervention and 4-6 weeks follow up.  -feedback forms | Knowledge level and retention quiz scores post-presentation and follow-up  were both significantly greater than baseline scores.  Positive feedback from students for this unique class presentation-interesting, open, interactive, knowledgeable. |
